# PDGF-responsive progenitors persist in the subventricular zone across the lifespan

**DOI:** 10.1042/AN20120041

**Published:** 2014-02-07

**Authors:** Lisamarie Moore, Jennifer M. Bain, Ji Meng Loh, Steven W. Levison

**Affiliations:** *Rutgers–New Jersey Medical School, Department of Neurology and Neurosciences, Newark, NJ 07103, U.S.A.; †Rutgers–Graduate School of Biomedical Sciences, Newark, NJ 07103, U.S.A.; ‡Rutgers–Cancer Center, Newark, NJ 07103, U.S.A.; §New Jersey Institute of Technology, Department of Mathematics, Newark, NJ 07102, U.S.A.

**Keywords:** astrocyte, central nervous system, neural stem cell, neuron, oligodendrocyte, rat, BMP-4, bone morphogenetic protein 4, DIV, days *in vitro*, DMEM, Dulbecco’s modified Eagle’s medium, EGF, epidermal growth factor, ERK, extracellular-signal-regulated kinase, FGF, fibroblast growth factor, GFAP, glial fibrillary acidic protein, MAPK, mitogen-activated protein kinase, NCS, newborn calf serum, NSC, neural stem cell, O-2A, oligodendrocyte-type 2 astrocyte, OPC, oligodendrocyte progenitor cell, PDGF, platelet-derived growth factor, PDGFR, PDGF receptor, PFA, paraformaldehyde, PRP, PDGF-responsive precursor, PSA-NCAM, polysialated neural cell adhesion molecule, SD, Sprague–Dawley, SHH, sonic hedgehog, SVZ, subventricular zone, T3, triiodothyronine

## Abstract

The SVZ (subventricular zone) contains neural stem cells and progenitors of various potentialities. Although initially parsed into A, B, and C cells, this germinal zone is comprised of a significantly more diverse population of cells. Here, we characterized a subset of postnatal PRPs (PDGF-AA-responsive precursors) that express functional PDGFα and β receptors from birth to adulthood. When grown in PDGF-AA, dissociated neonatal rat SVZ cells divided to produce non-adherent clusters of progeny. Unlike the self-renewing EGF/FGF-2-responsive precursors that produce neurospheres, these PRPs failed to self-renew after three passages; therefore, we refer to the colonies they produce as spheroids. Upon differentiation these spheroids could produce neurons, type 1 astrocytes and oligodendrocytes. When maintained in medium supplemented with BMP-4 they also produced type 2 astrocytes. Using lineage tracing methods, it became evident that there were multiple types of PRPs, including a subset that could produce neurons, oligodendrocytes, and type 1 and type 2 astrocytes; thus some of these PRPs represent a unique population of precursors that are quatropotential. Spheroids also could be generated from the newborn neocortex and they had the same potentiality as those from the SVZ. By contrast, the adult neocortex produced less than 20% of the numbers of spheroids than the adult SVZ and spheroids from the adult neocortex only differentiated into glial cells. Interestingly, SVZ spheroid producing capacity diminished only slightly from birth to adulthood. Altogether these data demonstrate that there are PRPs that persist in the SVZ that includes a unique population of quatropotential PRPs.

## INTRODUCTION

The SVZ (subventricular zone), most pronounced in the dorsolateral wall of the lateral ventricle, is comprised of a heterogeneous cell population consisting of NSCs (neural stem cells) and progenitors of various potentialities. Precursors isolated from the SVZ have been shown to be EGF (epidermal growth factor)- and FGF (fibroblast growth factor)-responsive (Reynolds et al., 1992; Reynolds and Weiss, 1992; Gritti et al., 1995, 1999), but more recent studies have determined that another subset of cells in the SVZ express receptors that respond to PDGF (platelet-derived growth factor) (Jackson et al., 2006; Assanah et al., 2009). PDGF has been shown to stimulate the growth of PDGFRα (PDGF receptor α)-positive cells from both the embryonic and the adult mouse SVZ (Chojnacki and Weiss, 2004; Jackson et al., 2006).

In the neonatal period, the SVZ is a major source of glial progenitors. These glial progenitors migrate from the SVZ ventro-laterally to the adjacent striatum and dorsally to the white matter and the neocortical gray matter, where they can give rise to both astrocytes and oligodendrocytes (Levison et al., 2005; Levison and Goldman, 2006; Zhu et al., 2008). Whereas the architecture and composition of adult SVZ has been richly described (Doetsch et al., 1997; Alvarez-Buylla and Garcia-Verdugo, 2002; Giachino et al., 2014), the newborn SVZ has received less attention, even though studies suggest that it is far more complex.

The newborn SVZ has been shown to contain unipotential astrocyte and oligodendrocyte progenitors as well as bipotential glial progenitors (Levison and Goldman, 1997). PDGFRα is generally agreed to be a factor essential for oligodendroglial development and has been shown to be expressed by neuroepithelial cells as early as E8.5 (Andrae et al., 2001). In the neonate, PDGFRα is expressed by oligodendrocyte precursors and their PSA-NCAM+ (polysialated neural cell adhesion molecule positive) pre-progenitors (Ben-Hur et al., 1998). These PSA-NCAM+ pre-progenitors have been isolated from P6 cerebral white matter by immunopanning and shown to generate oligodendrocytes and astrocytes *in vitro* (Grinspan et al., 1990a, 1990b). Upon characterization, these PSA-NCAM+ cells lacked markers for O-2A (oligodendrocyte-type 2 astrocyte) progenitors, such as GD3 and yet were able to give rise to O-2A cells that differentiated into type 2 astrocytes and oligodendrocytes (Grinspan et al., 1990a). Other studies established that PDGF is a survival factor for these PSA-NCAM+ pre-progenitors (Grinspan and Franceschini, 1995; Ben-Hur et al., 1998). When differentiated, they produced large percentages of oligodendrocytes and astrocytes, as well as a few neurons. However, clonal analyses were not performed to determine whether there was a common bipotential precursor or whether there were two sets of glial-restricted precursors that each expressed PDGFRα.

Some studies suggest that there are other multipotential precursors in the SVZ that are PDGFRα+. Although PDGFRα+ SVZ cells are generally associated with gliogenesis, there are PRPs (PDGF-responsive precursors) of the E14 ventral forebrain that are tripotential, giving rise to oligodendrocytes, astrocytes and neurons (Chojnacki and Weiss, 2004). Furthermore, in the adult brain, it has been reported that there is a subset of GFAP+ (glial fibrillary acidic protein) Type B cells that are also PDGFRα+ (Jackson et al., 2006). A more recent study, however, concluded that the PDGFRα+ precursors are not stem cells, and thus distinct from the GFAP+ adult stem cells of both mouse and human SVZ (Chojnacki et al., 2011).

Much of the research investigating PRPs of the SVZ have either focused on the glial-restricted precursors or multipotential cells of embryonic brain and some of these studies are contradictory (Jackson et al., 2006; Chojnacki et al., 2008; Jackson and Alvarez-Buylla, 2008; Chojnacki et al., 2011). To date, the PDGFRα+ cells of the neonatal SVZ have been poorly characterized. Therefore, the goal of this study was to investigate this interesting subset of SVZ cells. We sought to characterize their growth requirements, to determine whether they are stem cells or progenitors, to evaluate whether this is a homogeneous or diverse cell population and to assess their relative abundance across the lifespan.

## MATERIALS AND METHODS

### Spheroid cultures

Cultures were established from Wistar rat brains across a spectrum of ages, from postnatal day 3 (P3) to adult (P70) as well as from SD (Sprague–Dawley) rat pups that ubiquitously expressed GFP [Sprague-Dawley- Tg(GFP)Bal/2Rrrc (RRRC:0065) (Missouri Research Animal Diagnostics Laboratory)]. Newborn rats were decapitated under sterile conditions and their brains were placed into PBS with 0.6% glucose and 2 mM MgCl_2_. Adult rats were euthanized by carbon dioxide inhalation prior to decapitation. Incisions were made ~2 mm from the anterior end of the brain and ~3 mm posterior to the first cut. These blocks were transferred to fresh PBS-glucose-MgCl_2_ and the SVZ_DL_ and dorsal cerebral cortex were grossly isolated. Isolated tissue was minced with a scalpel and/or forceps. The tissue was then transferred to conical tubes and centrifuged at 200 ***g*** for 5 min. The pellet was enzymatically dissociated using a 2:3 dilution of Accutase (Innovative Cell Technologies) or an enzyme solution containing trypsin (0.25%), collagenase III (0.001 g), papain (0.01 g), DNase I (0.0002 g), MgSO_4_ (0.00385 g) and L-cysteine (0.0175 g) dissolved into 10 ml of MEM-Hepes. The neonatal tissue was digested for 5–10 min and adult tissue for 20 min at 37°C with manual agitation during incubation. An equal volume of medium supplemented to 10% NCS (newborn calf serum) was added and the mixture was triturated for several cycles using P1000 and P100 tips, adding additional media during later cycles. The single-cell suspension was passed through a 100 μm cell strainer and then a 40 μm strainer to eliminate clumped cells from the final mixture. Then the cells were centrifuged at 200 ***g*** for 5 min and the supernatant removed.

Viable cells were counted and plated at 3.75×10^4^, 7.5×10^4^ or 1.5×10^5^ cells/ml in ProN media [DMEM (Dulbecco's modified Eagle's medium)/F12 medium containing 10 ng/ml D-biotin, 25 μg/ml insulin, 20 nM progesterone, 100 μM putrescine, 5 ng/ml selenium, 50 μg/ml apo-transferrin and 50 μg/ml gentamycin (all purchased from Sigma–Aldrich, except gentamycin that was purchased from Life Technologies)]. ProN medium was supplemented with 30% B104 neuroblastoma conditioned medium (B104 CM) (Young and Levison, 1997), 10 ng/ml recombinant rat platelet-derived growth factor (PDGF-AA) (R&D Systems), 50 ng/mL noggin (R&D Systems) or 20 ng/ml EGF (Peprotech) and 5 ng/ml FGF-2 (Peprotech) with 1 ng/ml heparin sulfate (Sigma–Aldrich). PRPs were allowed to grow into spheroids for 7–10 DIV (days *in vitro*) in 2% O_2_, 5% CO_2_, 93% N_2_ or 20% O_2_, 5% CO_2_, 75% N_2_ at 37°C. To dissociate the spheroids, cells were either treated with Accutase or a pH-balanced solution containing collagenase III (0.002 g), papain (200 units), and DNase I (0.0002 g) in 10 ml of Papain Buffer [DMEM/F12 with 0.48 g of Hepes, 0.02 g of EDTA and 0.0175 g of L-cysteine]. Spheroids were continuously passaged by digestion in Accutase followed by mechanical trituration. Viable cells were plated at 7.5×10^4^ cells/ml in 50% spheroid conditioned media. To differentiate the spheroids, they were plated with or without dissociation in N2B2 media [ProN supplemented with 0.66 mg/ml BSA and either a 0.5% or 20% fetal bovine serum or 10 ng/ml BMP-4 (bone morphogenetic protein 4) (R&D Systems)] for 72 h. All animal work was performed according to Institutional Animal Care and Use Committee (IACUC) guidelines of UMDNJ, protocol #10012.

### Western blot

Spheroids (grown in ProN supplemented with PDGF-AA) and neurospheres (grown in ProN supplemented with EGF and FGF-2) were cultured for 7 DIV in 2% O_2_, 5% CO_2_ and 93% N_2_ as previously described (Alagappan et al., 2013). To evaluate responsiveness to PDGF, spheroids were growth-factor-starved for 6 h and then stimulated with 10 ng/ml PDGF-AA. Untreated spheroids were collected at 15 min and treated spheroids were collected at either 15 or 30 min after PDGF-AA addition. OPCs (oligodendrocyte progenitor cells) were generated from mixed glial cultures prepared from neonatal rat brains. Meninges were collected from the same animals prior to isolating deeper tissues. Cells were collected by gentle centrifugation and then lysed with 1% Triton-X100, 0.1% SDS, 1% 0.1 M sodium orthovanadate and protease inhibitor cocktail (Roche Diagnostics) dissolved in PBS. The cells were sonicated then centrifuged at 9300 ***g*** for 15 min at 4°C. The protein concentration was quantified with the Pierce BCA Protein Assay Kit (ThermoScientific). Twenty-five μg of denatured protein was loaded on to a 4–12% Novex NuPage Bis Tris Gel (Invitrogen) then transferred to nitrocellulose (Invitrogen). Blots were probed with rabbit anti-PDGFRα (1:1000, Cell Signaling Technology), rabbit anti-PDGFRβ (1:1000, Cell Signaling Technology), rabbit anti-phospho-p44/42 MAPK (mitogen-activated protein kinase) [pERK1/2 (extracellular-signal-regulated kinase 1/2)] (1:1000, Cell Signaling Technology), rabbit anti-p44/42 MAPK (ERK1/2) (1:1000, Cell Signaling Technology), and mouse anti-β-actin (1:5000, Sigma–Aldrich), washed and incubated with secondary antibody against rabbit or mouse conjugated to HRP (horseradish peroxidase) (1:2500, Jackson ImmunoResearch Laboratories). Signals were detected by chemiluminescence-ECL (PerkinElmer) and quantified using a UVP Bioimaging system.

### Immunofluorescence

Cells were fixed with 4% PFA (paraformaldehyde) and stained with primary antibodies for 1 h at room temperature (20°C) or overnight at 4°C, using mouse anti-PSA-NCAM (1:500, Millipore), O4 (1:5, supernatant), A2B5 (1:4, supernatant), D1.1 (1:4, supernatant), R24 (1:4, supernatant), Rmab (1:3, supernatant), TuJ1 (1:400, Covance), rabbit anti-NG2 (1:200, generously provided by Bill Stallcup), rabbit anti-Olig2 (1:500, Millipore) and rabbit anti-GFAP (1:500, Dako) antibodies.

For additional differentiation experiments, single cells (2–4×10^4^ cells/well) or whole spheroids (10–20 spheroids/well) were plated on to PDL and laminin-coated chamber slides and fed with N2B2 media or N2B3 media [N2B2 with 30 nM T3 (triiodothyronine)] and 2% NCS and maintained in an atmospheric incubator overnight to allow cells to adhere to the slides. The differentiation media was replaced with either N2B2+0.5% FBS, N2B3+0.5% FBS or N2B2+0.5% FBS supplemented with 10 ng/ml human BMP-4 (R&D Systems) for 3–5 DIV. To identify astrocytes, oligodendrocytes and neurons, cells were stained live for O4, Rmab, or A2B5 for 1 h at room temperature, rinsed and incubated with appropriate fluorochome-conjugated secondary antibodies for 1 h at room temperature. The cells were fixed for 5 min in 4% PFA and sometimes 100% methanol, blocked, and then incubated with TuJ1 and GFAP. The cells were washed and further incubated with secondary antibodies. Some cells were counterstained with DAPI for 10 min.

Stained cells were washed thoroughly and mounted with Fluorogel (EMS) and allowed to dry overnight. Immunoreactive cells were visualized using an Olympus Provis AX70 microscope and images of stained cells were collected using a Photometrics cooled charged coupled device camera interfaced with IP Lab scientific imaging software (Scanalytics). Labeled cells in at least four random (non-adjacent) fields were counted per well under a 20× or 40× objective and a total of four wells per independent group were evaluated with at least 100 cells counted based on DAPI staining. To evaluate the potentiality of a single spheroid, a minimum of 60 spheroids per group were assessed for their potentially.

### pNIT retrovirus production, cell infection and clonal analysis

Replication-deficient viruses with VSV-g coats were generated from pNIT- plasmid (Milosevic and Goldman, 2002) as previously described (Bain et al., 2010). Briefly, pNIT-293 cells were maintained with 0.8 mg/ml G418 in DMEM with 10% NCS. Cells were split on to seven PDL-coated plates in media lacking G418 2–3 days before transfection. When pNIT-293 cells were 90–95% confluent, a VSV-g plasmid was transfected into the cells using Lipofectamine™ 2000 Reagent (Life Technologies). Supernatant from transfected virus producing cells were collected after 2–3 days and titered by incubating NIH 3T3 cells with serial dilutions of retrovirus. The CFU was calculated by multiplying the number of GFP+ cell clusters by the dilution factor.

SVZ cells from Wistar neonates were propagated as spheroids and then gently dissociated into a single cell suspension and seeded at 2×10^4^ cells/well on to PDL-coated chamber slides in ProN with or without B104 CM supplemented with 2% serum and maintained in an atmospheric incubator for 6–12 h. Dissociated cells were infected with 50 CFU pNIT retrovirus followed by medium replacement 24 h later to N2B2+0.5% FBS. Media was changed every other day for 7 days, after which cells were fixed in 4% PFA and stained for GFP (Aves Labs), O4, GFAP and TUJ1.

In a separate set of experiments, SVZ cells from GFP-expressing SD rats were dissociated and mixed with SVZ cells isolated from wild-type SD rats at an initial cell ratio of 1:150, 1:75, 1:30 and 1:15. These cells were propagated as spheroids and then seeded as whole spheroids (~30 spheres/well) on to PDL and laminin-coated chamber slides in ProN with 2% serum. Media (N2B2+0.5% FBS) was changed every other day for 7 days, after which cells were fixed in 4% PFA and stained for GFP, O4, GFAP and TUJ1.

### Limiting dilution assay

Primary spheroids and neurospheres were cultured as previously described above, dissociated and plated into a 96-well plate with a minimum of six replicate wells per condition. Cells were plated at 1000, 750, 500, 250, 100, and 50 cells per well in 200 μl of ProN supplemented with 10 ng/ml PDGF-AA or 20 ng/ml EGF and 10 ng/ml FGF-2 with 50% conditioned media. After 7 DIV wells were scored for the presence of at least one sphere per well. To discern a sphere from a cluster of cells the cell suspension was pipetted several times and then analyzed. To measure the fraction of sphere-forming cells where μ equals the number of positive wells and r equals the total number of wells, the Poisson discrete distribution probability 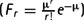 was used where negative wells (*F_o_*=*e*^− μ^) and positive wells (*F*_1_=μ*e*^− μ^) are plotted as a semi log plot (Lefkovits and Waldmann, 1999).

### Statistical analysis

Results were analyzed for statistical significance using a Student's *t* test or by ANOVA with Fisher's PLSD post-hoc test. Error bars represent S.E.M. Comparisons were interpreted as significant when associated with *P*<0.05.

## RESULTS

The postnatal SVZ is a mosaic of precursors that have the potential to produce many different types of neurons and glia of the nervous system. The neurosphere culture method provides the means to propagate NSCs as well as their progeny; however, there are other precursors residing in the SVZ that have been less well studied. Importantly, to date there is no accepted cell culture system for growing the tripotential and bipotential progenitors of the SVZ. Here we specifically sought to optimize an *in vitro* system to culture PRPs and to characterize their developmental potential.

To propagate PRPs, the SVZs of neonatal rats were microdissected from the brain, dissociated into a single-cell suspension and grown in a hormone-supplemented medium containing 10 ng/ml PDGF-AA. Over several days *in vitro* these precursors divided and formed clonal clusters that grew independent of the substrate, like the neurospheres formed by NSCs. However, these colonies are distinct from neurospheres; therefore, we will refer to these clusters as spheroids. For example, the spheroids were less compact than the conventionally studied neurospheres.

**Figure 1:**
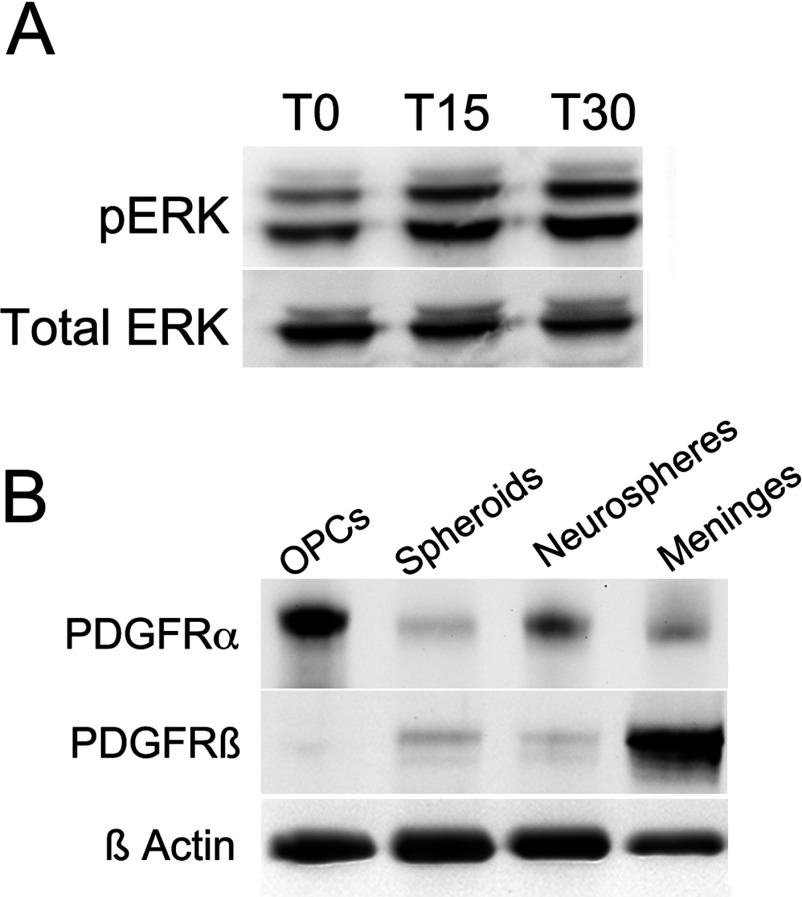
Spheroids are responsive to PDGF-AA and express PDGFRα and PDGFRβ Spheroids and neurospheres were generated from neonatal SVZs and cultured for 7 DIV in 2% O_2_, 5% CO_2_, 93% N_2_. OPCs were generated from mixed glial cell cultures from neonatal rat brains. (**A**) Expression levels of pERK1/2 (44,42 kDa) and total ERK1/2 (44,42 kDa) in 6 h growth factor-starved spheroids untreated (T0) or treated with 10 ng/ml PDGF-AA stimulation for 15 min (T15) and 30 min (T30). (**B**) Expression levels of PDGFRα (195 kDa) and PDGFRβ (185 kDa) in OPCs, spheroids, neurospheres and meninges. Data are representative of two independent experiments.

Levels of phosphorylated ERKs were measured to determine whether the PRPs within the spheroids were responsive to exogenous PDGF-AA. After a 6 h starvation period (T0), pERK-1 and 2 were detected as a doublet that migrated at 44 and 42 kDa (Figure 1A). Upon addition of PDGF-AA to achieve 10 ng/ml in the media, levels of both pERK-1 and 2 increased above baseline by 15 min (T15) and 30 min (T30).

To determine whether the cells in the spheroids expressed either or both the α or β isoforms of the PDGFRs, Western blotting was performed using well-characterized antibodies against these receptors (Figure 1B). OPCs and meningeal fibroblasts were used as positive controls for the PDGFRα and PDGFRβ, respectively. A band that migrated at 195 kDa was seen using the antibody for PDGFRα. The relative expression levels for the PDGFRα were as follows, OPCs>neurospheres>meninges>spheroids. A band that migrated at 185 kDa was seen using the antibody for PDGFRβ. The expression of this receptor differed from that of the PDGFRα with expression as follows: meninges>spheroids>neurospheres>OPCs. These studies verified that the PRPs do indeed express PDGFRα and lower levels of PDGFRβ.

Cells propagated as spheroids were characterized using a panel of stem, progenitor and lineage-restricted antigens (Table 1). The majority of cells within a spheroid were PSA-NCAM+ (78%), while 39% expressed the oligodendrocyte transcription factor 2 (Olig2). About one-third expressed nestin (34%) and GFAP was present in 21%. Of the markers that label O-2A cells, 19.1% and 17.5% were immunoreactive for A2B5 and R24 surface antigens, and 26% expressed the NG2 chondroitin sulfate proteoglycan. Less than 10% labeled using the D1.1 monoclonal antibody.

**Table 1:** Markers expressed by acutely dissociated cells from glial spheroids Spheroids were grown for 7–10 days in B104 CM in 2% O_2_, 5% CO_2_, 93% N_2_ and gently dissociated into single cells. Cells were cytospun on to PDL-coated chamber slides at 2×10^4^ cells/well and then immediately fixed and stained. Cells were analyzed under 20× or 40× magnification for the presence or absence of markers. Data represents averages of three independent experiments.

Antigen	Total cells (%)
PSA-NCAM	77.5
Olig2	38.7
Nestin	34.8
NG2	25.9
GFAP	21.0
Tetrasialoganglioside (A2B5)	19.1
GD3 ganglioside (R24)	17.5
Acetylated GD3 ganglioside (D1.1)	9.4

**Figure 2:**
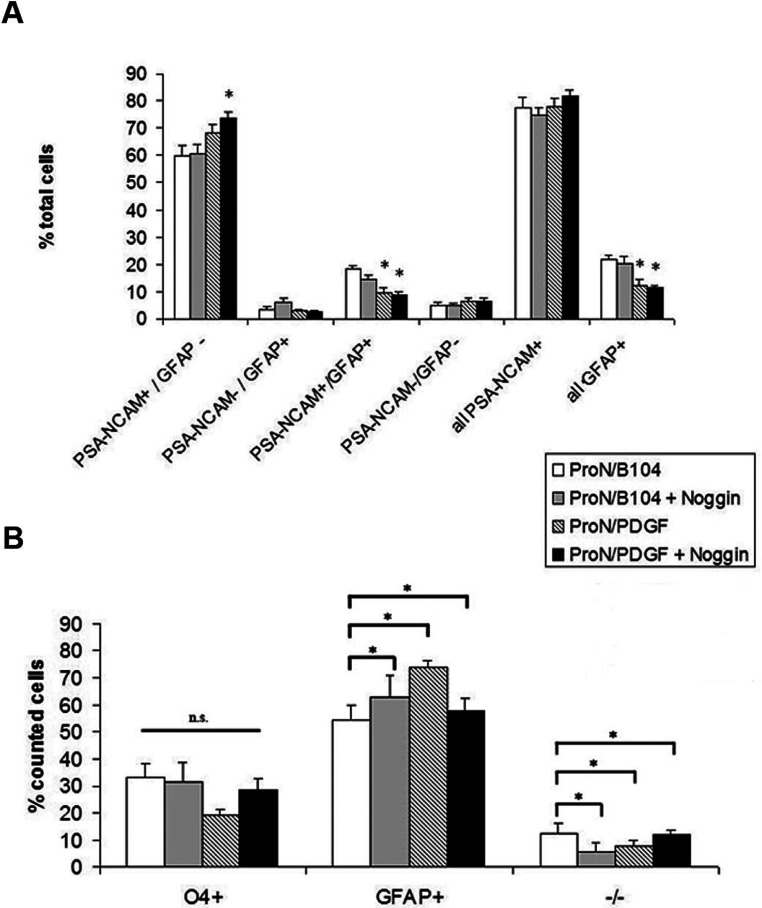
Expression of PSA-NCAM and GFAP in spheroids and differentiation into immature oligodendrocytes and astrocytes under different growth conditions (**A**) Spheroids were gently dissociated and plated on to PDL-coated chamber slides for PSA-NCAM and GFAP immunostaining. (**B**) Spheroids were dissociated into single cells, plated for 6 h with 2% serum and grown in an atmospheric incubator. Media was changed to N2B2 with 0.5% serum for 96 h. Cells were then stained for O4, followed by fixation and then for GFAP and DAPI. Data represent averages±S.E.M. from three independent experiments. * denote significant differences in (**A**) versus Pro-N/B104. n.s. denotes no statistical significance. For comparisons in both (**A**) and (**B**), *P*<0.05 using ANOVA followed by Fisher's PLSD post-hoc test.

As we were interested in creating a culture system favorable for studying early progenitors, and as PSA- NCAM has been reported to be expressed on early neural and glial progenitors, we evaluated several medium formulations for their ability to enrich for PSA-NCAM+ cells (Figure 2A). The basal media for all cultures was a hormone-supplemented, serum-free media. One test medium was supplemented with B104 CM±noggin, a BMP antagonist. BMPs have been previously established to contribute to astrocyte specification (Mabie et al., 1997; Grinspan et al., 2000); therefore, noggin was added to neutralize any BMPs that might be generated by the progenitors. The other medium formulation was supplemented with 10 ng/ml PDGF-AA.

Contrary to our hypothesis, the addition of noggin had no effect on spheroid formation. Furthermore, we found no significant difference in the total percentage of PSA-NCAM+ cells, regardless of the growth conditions, with the average percentage ranging from 74.9% to 82% PSA-NCAM+ cells (Figure 2A). The relative GFAP+ cell percentages ranged from 11.1% to 21.6% among groups. To assess the differentiation potential, the spheroids were dissociated and differentiated for 4 days. All four conditions generated similar ratios of type 1 astrocytes to oligodendrocytes, with low levels of O4−/GFAP− cells (Figure 2B).

**Figure 3:**
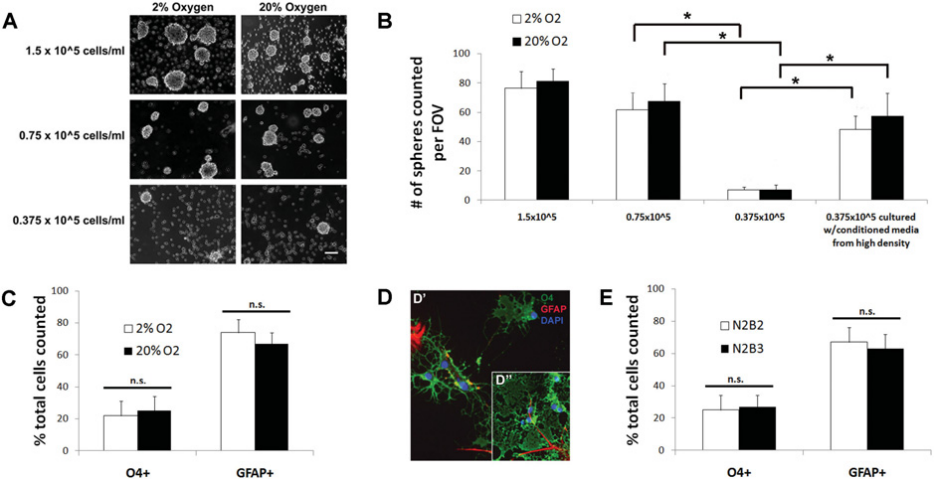
Cell density affects spheroid generation while O_2_ tension affects oligodendrocyte differentiation, however neither O_2_ nor T3 affect glial specification (**A**) Cultures were seeded at initial densities of 1.5×10^5^, 0.75×10^5^ and 0.375×10^5^ cells/ml and grown for 8–10 days in 2% O_2_ or 20% O_2_ in B104 CM. (**B**) Quantification of the average number of spheroids per field of view (FOV). (**C**) Cells were grown in 2% O_2_ and differentiated in either 2% O_2_ or 20% O_2_, then analyzed for positive staining of oligodendrocytes (O4) and astrocytes (GFAP+). (**D**) Fluorescence images of the cells differentiated in 2% O_2_ (**D**’) or 20% O_2_ (**D**”). (**E**) Cells were grown in 2% O_2_ and differentiated in the absence of T3 (N2B2) or presence of T3 (N2B3) at 20% O_2_. Scale bar represents 100 μm (**A**) and 10 μm (**D**). Error bars represent±S.E.M. from at least three independent experiments. * denote significant differences and n.s. denotes no statistical significance, where *P*<0.05 using Student's *t* test.

The effect of the initial plating cell density on the number of spheroids formed was evaluated since cells secrete growth and trophic factors that conditions the medium and, therefore, density may affect their growth. Spheroids were plated at initial densities of 1.5×10^5^ cells/ml, 7.5×10^4^ cells/ml and 3.75×10^4^ cells/ml and maintained for 10 days (Figure 3A). These studies showed that cell density was crucial for primary spheroid development. There was a non-linear decrease in spheroids formed at the lowest cell density (0.375×10^5^ cells/ml) within both O_2_ conditions (Figure 3B). Interestingly, when cells plated at the lowest density were provided conditioned media from the cells cultured at the highest density there was a robust recovery of spheroid formation. In the following experiments cells were plated at an initial density of 0.75×10^5^cells/ml.

Extracellular O_2_ and intracellular reactive oxygen species can regulate the balance between self-renewal and differentiation in neural precursors (Smith et al., 2000; Noble et al., 2003; Le Belle et al., 2011); therefore, superphysiological levels of O_2_ could affect the proliferation and differentiation of the spheroids. To determine whether O_2_ had an effect on rat spheroid growth, neonatal rat SVZ cells were cultured in either 2% O_2_ or 20% O_2_ (Figure 3A). Spheroids were generated in all culture conditions and there was no significant difference in spheroid numbers as a function of O_2_ level (Figure 3B). However, spheroids grown in 2% O_2_ were generally larger than those grown in 20% O_2_ (Figure 3A). In subsequent experiments cultures were maintained in 2% O_2_.

To determine whether O_2_ would affect macroglia maturation, spheroids generated in 2% O_2_ were differentiated in either 2% or 20% O_2_ for 5 days. In these experiments there was no significant difference in the percentages of O4+ or GFAP+ cells differentiated in 2% or 20% O_2_ (Figure 3C). However, the morphology of the differentiated O4+ cells varied with O_2_ concentration. Oligodendrocytes formed when differentiated in 2% O_2_ possessed fine processes and fewer myelin-like sheets (Figure 3D’) and, therefore, may be less mature than the oligodendrocytes differentiated in 20% O_2_ that displayed a more mature morphology (Figure 3D”). In general the SVZ is not a heavily vascularized region, therefore, the lower O_2_ conditions may sustain cells in a less differentiated state. In subsequent experiments, cells were allowed to differentiate in 20% O_2_.

**Figure 4:**
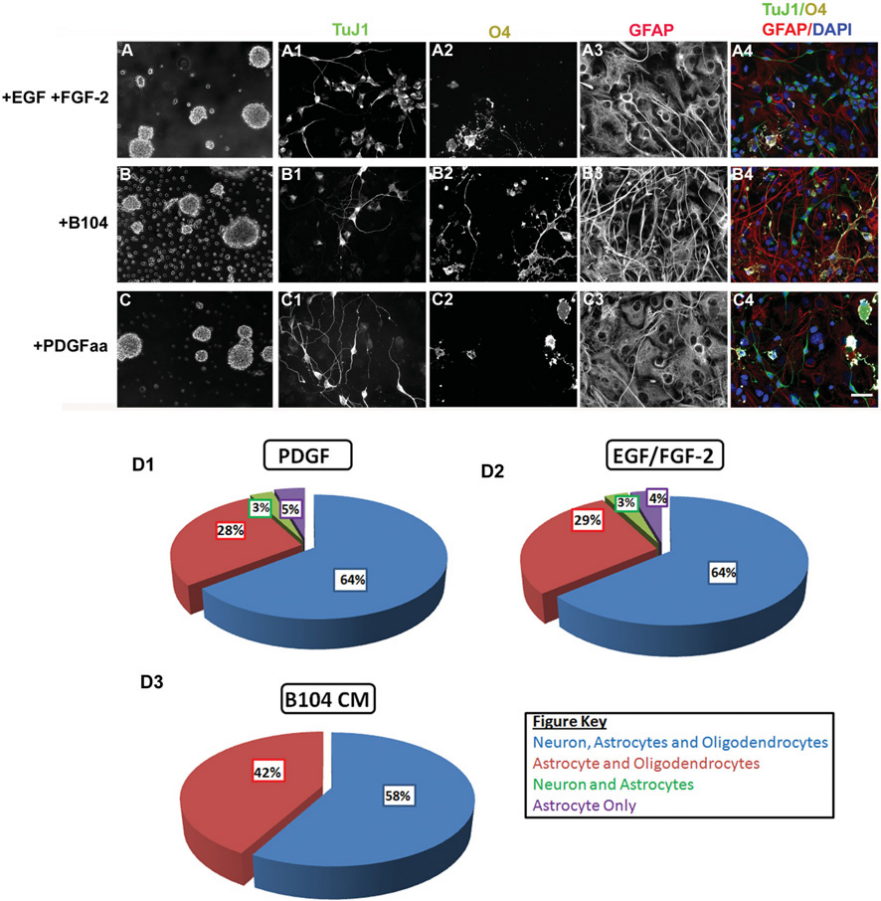
Greater than half of the spheroid-forming precursors are tripotential Neurospheres (**A**) and spheroids (**B** and **C**) were grown for 8–10 days in 2% O_2_ ProN medium supplemented with 10 ng/ml EGF and 5 ng/ml FGF-2, 30% B104 CM, or 10 ng/ml PDGF-AA. The spheres were plated on to slides and differentiated for 5 days then stained for neuronal (TUJ1) (**A1**, **B1**, **C1**), oligodendrocyte (O4) (**A2**, **B2**, **C2**), and astrocyte (GFAP) (**A3**, **B3**, **C3**) markers. The pseudocolor images show an overlay of TUJI (green), O4 (white), GFAP (red), and DAPI (blue) (**A4**, **B4**, **C4**). At least 60 individual neurospheres (**D1**) and spheroids **(D2**, **D3)** were analyzed based on colony composition. Pie charts represent three independent experiments. Scale bar represents 100 μm (**A**, **B**, **C**) and 10 μm (**A1**–**A4, B1**–**B4, C1**–**C4**).

Several studies have provided thyroid hormone to enhance oligodendrocyte production from glial precursors (Baas et al., 1997; Jones et al., 2003; Chojnacki and Weiss, 2004). Therefore, we added thyroid hormone (T3) to the spheroid growth medium; however, no significant difference in the percentage of O4+ cells in the presence or absence of T3 was observed (Figure 3E). Similarly, the ratio of cells that differentiated into immature oligodendrocytes (O4+) or type 1 astrocytes (GFAP+) remained constant. In subsequent experiments cells were differentiated in the absence of T3. Population studies have provided evidence for the existence of embryonic PRPs that can generate both neurons and glia, whereas postnatal PRPs have been generally regarded as glial restricted precursors. Therefore, we sought to determine whether the postnatal SVZ PRPs were multipotential (Figure 4). SVZ cells were cultured in media containing EGF/FGF-2, B104 CM or PDGF-AA. After 8–10 DIV the neurospheres (Figure 4A) or spheroids (Figures 4B and 4C) were differentiated for 5 days in the absence of growth factors on PDL and laminin-coated chamber slides. All three culture media supported macroglia growth as expected (Figures 4A2, 4B2, 4C2, 4A3, 4B3 and 4C3). Unexpectedly, B104 CM and PDGF-AA supplemented media also produced TUJI+ neurons as did the EGF/FGF-2 responsive NSCs (Figures 4A1, 4B1 and 4C1). In fact, when individual neurospheres were analyzed, 64% had at least one neuron present although the bulk of cells were oligodendrocytes and astrocytes (Figure 4D2). Similarly, the majority of spheroids (Figures 4D1 and 4D3) were tripotential. Interestingly, the EGF/FGF-2 responsive neurospheres and PDGF-AA responsive spheroids had almost identical differentiation ratios where 64% were tripotential, approximately 30% were bipotential producing astrocytes and oligodendrocytes, 3% were bipotential producing neurons and astrocytes, and approximately 5% were unipotential restricted to only astrocyte production (Figures 4D1 and 4D2). The neurospheres and spheroids that only contained astrocytes were significantly smaller than the spheres or spheroids that were tri- or bipotential. We did not observe any bipotential spheroids containing neurons and astrocytes or unipotential spheroids when cultured in B104 CM (Figure 4D3).

**Figure 5:**
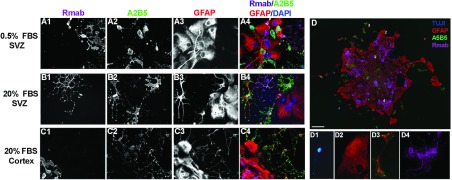
Spheroids from the neonatal SVZ and neocortex generate colonies that contain neurons, type 1 astrocytes, type 2 astrocytes and oligodendrocytes Spheroids were cultured with 10 ng/ml PDGF-AA, differentiated for 3 days in either 0.5% FBS or 20% FBS then stained for oligodendrocytes (Rmab) (**A1**, **B1**, **C1**), O-2A lineage (A2B5) (**A2**, **B2**, **C2**), and astrocytes (GFAP) (**A3**, **B3**, **C3**). The pseudocolor images show an overlay of Rmab (magenta), A2B5 (green), GFAP (red), and DAPI (blue) (**A4**, **B4**, **C4**). When differentiated in 0.5% FBS and 10 ng/ml BMP-4, a single spheroid produced neurons (TUJ1), type 1 astrocytes (A2B5−/GFAP+), type 2 astrocytes (A2B5+/GFAP+) and oligodendrocytes (Rmab) (**D**). Examples of the four cell types from a single spheroid neuron (**D1**), type 1 astrocyte (**D2**), type 2 astrocyte (**D3**) and oligodendrocyte (**D4**) within the spheroid. Scale bar represents 20 μm.

We next sought to determine whether the spheroids could also produce type 2 astrocytes. For these experiments spheroids from the SVZ and neocortex were grown and differentiated in either 0.5% or 20% FBS (Figure 5). After 3 days, cells were stained for the oligodendrocyte marker, Rmab (Figures 4A1, 4B1 and 4C1), the O-2A cell and type 2 astrocyte marker A2B5 (Figures 5A2, 5B2 and 5C2) and the intermediate filament protein of both type 1 and type 2 astrocytes, GFAP (Figures 5A3, 5B3 and 5C3). All conditions produced Rmab+ oligodendrocytes and A2B5−/GFAP+ type 1 astrocytes. Confirming our predictions, there were few, if any, A2B5+/GFAP+ type 2 astrocytes when differentiated in the presence of 0.5% FBS (Figure 4A4), whereas there was a robust production of type 2 astrocytes from the SVZ (Figure 5B4) and neocortical (Figure 5C4) spheroids when differentiated in 20% FBS.

Although a high serum concentration is optimal for type 2 astrocyte differentiation, serum is toxic for neurons *in vitro*. Therefore, to verify that a single spheroid could produce neurons, oligodendrocytes, type 1 astrocytes and type 2 astrocytes we lowered the concentration of serum to 0.5% FBS and supplemented the medium with 10 ng/ml BMP-4, to simultaneously promote neuron differentiation and type 2 astrocyte production. These culture conditions permitted a single spheroid to produce all four cell types (Figure 5D). In fact, the majority of spheroids from the neonatal SVZ were capable of producing neurons (Figure 5D1), type 1 (Figure 5D2) and type 2 astrocytes (Figure 5D3), and oligodendrocytes (Figure 5D4). By contrast, although spheroids could be generated from the neonatal neocortex, neurons were rarely produced from neocortical spheroids.

**Figure 6:**
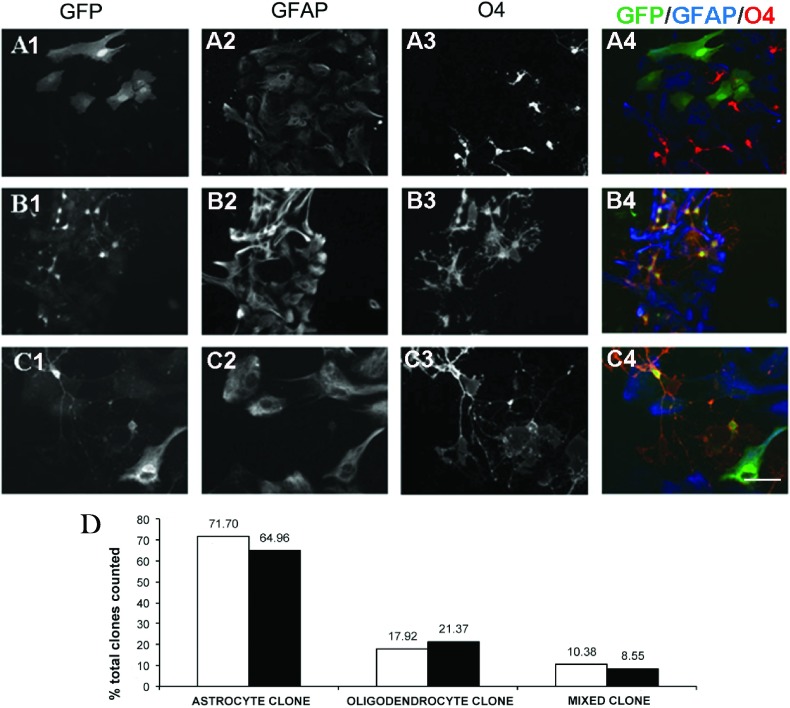
Clonal analyses of spheroids reveal three subclasses of progenitors Spheroids were grown for 7–10 days in 2% O_2_, 5% CO_2_, 93% N_2_ and then gently dissociated into single cells and plated on to PDL-coated chamber slides. Cells were infected with 50 CFU pNIT retrovirus for 1 day followed by growth in N2B2+0.5% FBS for 7 days after which cells were fixed in 4% PFA and triple-stained for GFP-green (**A1**, **B1**, **C1**), GFAP-blue (**A2**, **B2**, **C2**) and O4-red (**A3**, **B3**, **C3**). Clones were classified as astrocyte - only clones (**A4**); oligodendrocyte - only clones (**B4**); or mixed clones (**C4**). Scale bar represents 40 μm (**A** and **B**) and 20 μm (**C**). (**D**) Quantification of two independent experiments in which clonal analyses were performed after infection with 1×10^5^ CFU of the pNIT reporter replication-deficient retrovirus in N2B2 differentiation media (white) or in ProN/B104 CM (black).

Whereas the spheroids produced neurons, astrocytes and oligodendrocytes, these spheroids might be comprised of lineage-restricted progenitors, with separate precursors for astrocytes and others for oligodendrocytes or neurons, or they might be comprised of multipotential progenitors that could give rise to all cell types. To characterize the differentiation potential of individual progenitors we used replication-deficient retroviruses and infected a limited number of cells. The fates of cells derived from single precursors were then analyzed after 5 days of differentiation (Figure 6). A colony was defined as GFP+ cells that were at least 150 μm from another cluster. This technique has been previously established to define single clones while avoiding the overlapping of two separate clones (Levison and Goldman, 1993, 1997). Figures 6A1–6A4 depict a clone that contained only type 1 astrocytes. Figures 6B1–6B4 depict a colony that was comprised of oligodendrocytes and Figures 6C1–6C4 depict a colony that was comprised of both type 1 astrocytes and oligodendrocytes. In one experiment, the cells were infected during differentiation and 109 clones were analyzed (average of 5.5 clones per well). Of these colonies, 72% were astrocyte-generating, 18% were oligodendrocyte-generating and 10% generated mixed colonies containing both astrocytes and oligodendrocytes. In a follow-up experiment, where the cells were infected while proliferating in the B104 CM, a strikingly similar outcome was obtained. Out of a total of 117 counted colonies, 65% were astrocyte-generating, 21% oligodendrocyte-generating and 8% generated mixed glial colonies. Contrary to our previous experiments where many TUJ1+ neurons were observed, TUJI+/GFP+ neurons were not observed in either experiment, even though TUJI+/GFP− neurons were present in the cultures.

**Figure 7:**
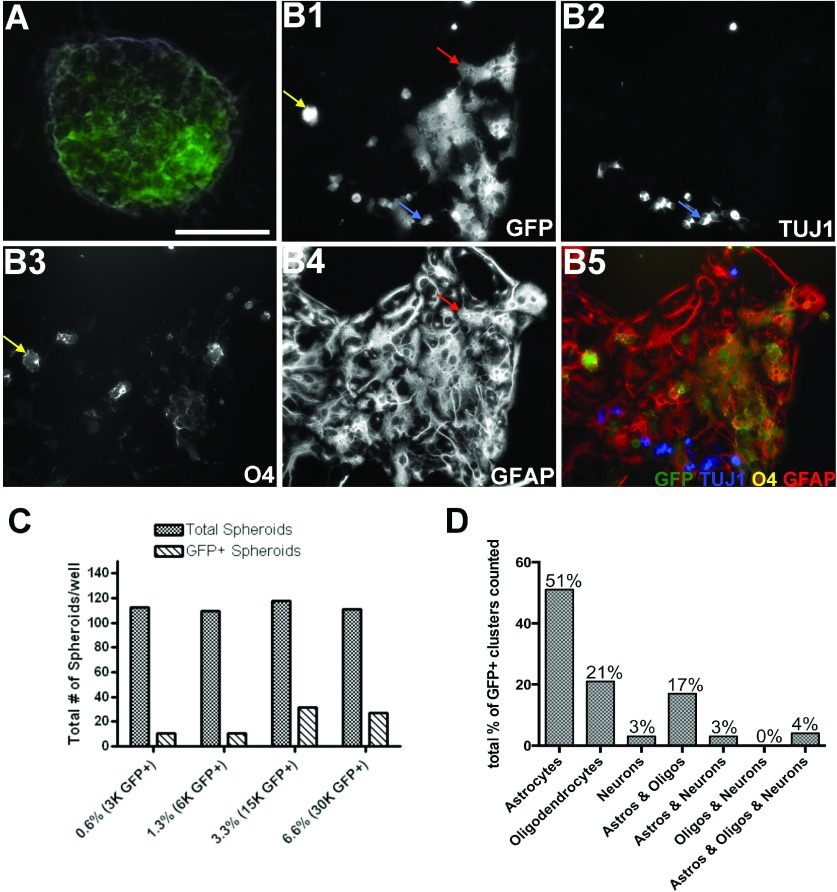
*In vitro* co-cultures reveal probability and potentially of single PDGF-responsive SVZ cells SVZs from P3, GFP+/− and GFP−/−, littermates were grown at a low density of 5×10^4^ cells/ml in co-culture at a ratio of 1:150 for 8 days in serum-free preconditioned media and atmospheric gases of 2% O_2_, 5% CO_2_, 93% N_2_. Most spheroids were comprised of both GFP+ and GFP− cells (**A**). Spheroids were then plated on to PDL and laminin-coated chamber slides in the absence of growth factors and differentiated for 5 days. Clusters of GFP+ cells (**B1**) were analyzed for antigens TUJ1 (**B2**), O4 (**B3**) and GFAP (**B4**). Spheroids that contained ≥5 GFP+ cells, by fluorescence imaging followed by ImageJ analysis, were counted as a GFP+ spheroids (**C**). Colonies of GFP+ cells were classified as astrocyte-only, oligodendrocyte-only, neuron-only, astrocyte-oligodendrocyte, astrocyte-neuron, oligodendrocyte-neuron, astrocyte-oligodendrocyte-neuron (**D**). Scale bar represents 40 μm (**A**).

Since specific types of neurons are known to silence retroviral vector reporter gene expression (Gaiano et al., 1999), it is possible that neurons produced from a PRP may have silenced GFP expression and thus eluded detection. Therefore, we used a co-culture system where SVZ cells were isolated from the SVZs of P3 transgenic GFP+ neonates and their GFP− littermates, and then mixed whereupon the GFP+ cells comprised a small proportion of the total. Single cells were suspended at ratios of 1:150, 1:75, 1:30, and 1:15 (GFP+/GFP−) and cultured in 50% conditioned media. After 8 DIV, spheroids containing a mixture of GFP+ and GFP− cells were frequently observed (Figure 7A); however, small spheroids comprised uniformly of GFP+ cells were also observed. Spheroids were scored as GFP+ if they contained at least five or more GFP+ cells, even if some of the cells in the spheroid were GFP− (Figure 7C).

**Figure 8:**
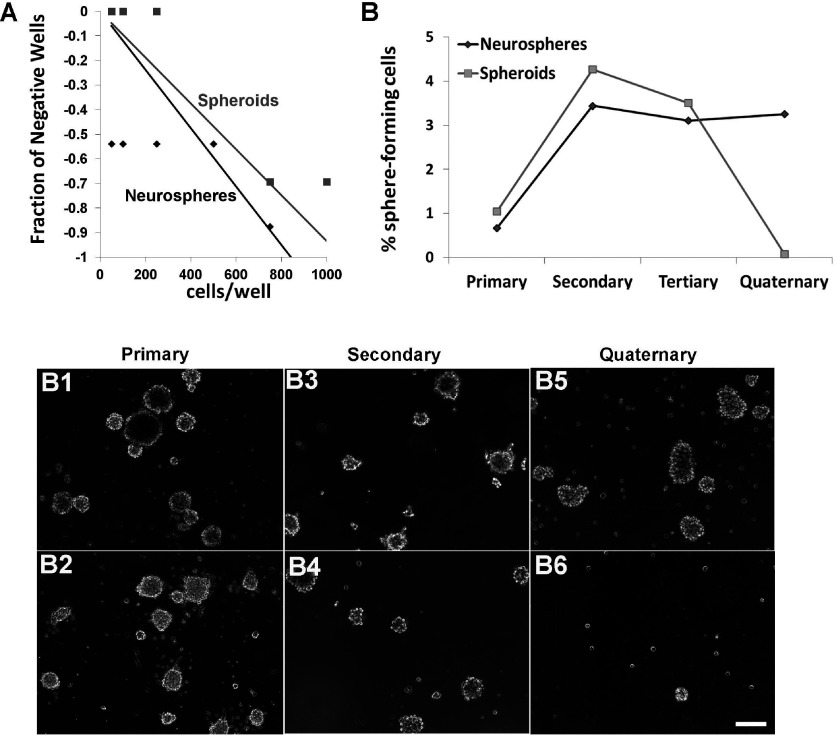
Neurospheres contain a greater number of self-renewing cells than spheroids, whose precursors lose their self-renewal capacity over serial passages (**A**) Spheroids and neurospheres were generated from neonatal SVZs and cultured for 7 DIV in 2% O_2_, 5% CO_2_, 93% N_2_, passaged and plated in a 96-well plate. The limiting dilution assay was performed by plating cells at decreasing densities and then scoring the fraction of negative wells. The reciprocal of the natural logarithm (*e*) was used to measure the frequency of sphere-forming cells in neurospheres (black) and spheroids (gray). (**B**) Spheroids and neurospheres were dissociated into single cells and re-plated every 7 days to generate subsequent spheres. The percentage of sphere-forming cells to the total cells plated was determined. Representative images of neurospheres and spheroids from primary (**B1**, **B2**), secondary (**B3**, **B4**) and quaternary (**B5**, **B6**) spheres. Data represent the averaged from two independent experiments.

Since many of the spheroids contained GFP+ and non-GFP+ cells, that may have formed by PRP adhesion or spheroid fusion, we performed a statistical analysis using the hypergeometric probability model to determine the probability that the GFP+ cells within a spheroid were descended from a single progenitor (Ross, 2010). Given the number of GFP+ to GFP− cells we initially plated (3×10^3^ GFP+ to 4.5×10^5^ GFP−) and the generation of 112 spheroids, of which 11 were GFP+ (containing at least five GFP+ cells), it was possible to compute the probability that a single GFP+ PRP formed a cluster of GFP+ cells within the spheroid. The model concluded that for there to be a greater than 5% chance that more than two GFP+ PRPs are giving rise to daughter cells in a single spheroid, the number *k* of spheroid forming cells within a single spheroid would have to exceed 15 parent cells. Although *k* is unknown, *k* exceeding 15 is highly unlikely based on our limited dilution assay where the percentage *r* of spheroid-forming cells is approximately 1% (Figure 8B). Therefore, we can conclude that in our lowest plating density of GFP+ cells (1:150) there were ~30 GFP+ PRPs (*r*=1% of 3×10^3^) that were spheroid forming. These 30 GFP+ PRPs comprised a mosaic of over 4.5×10^5^ cells where the vast majority were GFP−. Thus, the likelihood of two or more GFP+ PRPs adhering to each other in these culture conditions are highly improbable and, therefore, we can report with almost certainty that each GFP+ cluster originated from one GFP+ cell.

To assess the developmental potentials of these PRPs, all of the spheroids from the lowest initial plating density of GFP+ cells were plated on to PDL and laminin-coated chamber slides and differentiated in the absence of growth factors or serum for 5 days. Clusters of GFP+ cells that were widely separated from each other were stained for TUJ1 (Figure 7B2), O4 (Figure 7B3), and GFAP (Figure 7B4). We counted 90 colonies and found that 51% of the clones were astrocyte-generating, 21% were oligodendrocyte-generating, 3% were neuron-generating, 17% were bipotential astrocyte and oligodendrocyte generating, and 3% were bipotential astrocyte and neuron generating. None produced oligodendrocytes and neurons and 4% were tripotential generating astrocytes, oligodendrocytes and neurons (Figure 7D).

**Figure 9:**
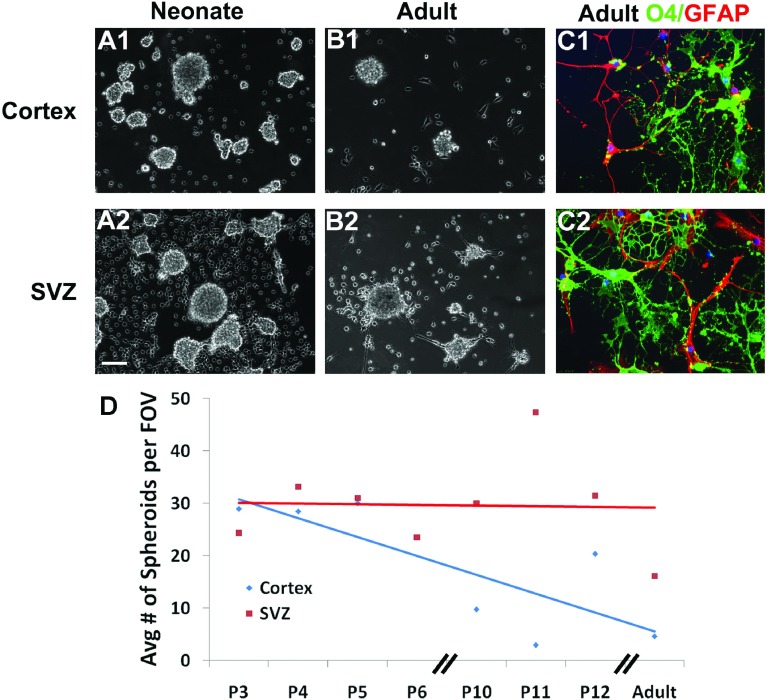
Neocortical spheroid formation decreases with age Spheroids were generated from P5 neocortex (**A1**), P5 SVZ (**A2**), P70 neocortex (**B1**) and P70 SVZ (**B2**). Neonatal cells were grown for 7 days while the adult cells where grown for 16 days in 2% O_2_ in B104 CM. Spheroids were plated on to slides and differentiated for 5 days then stained for oligodendrocyte (O4-green) and astrocyte (GFAP-red) markers (**C1**, **C2**). The average number of spheroids from P3, P4, P5, P6, P10, P11, P12 and adult (P64–P70) rodents is plotted (**D**). Scale bar represents 100 μm (**A1**–**A2**, **B1**–**B2**) and 10 μm (**C1**–**C2**).

Jackson et al. (2006) suggested that some PDGFRα+ cells in the adult SVZ were stem cells. Therefore, to determine whether the spheroids were formed by stem cells we tested their self-renewal capability (Figure 8). To determine the number of cells within a sphere that were self-renewing and capable of giving rise to subsequent spheres we used the limiting dilution assay. Using informative serial dilutions we determined the ratio of sphere-forming cells within spheroid and neurosphere cultures. To graphically represent the Poisson distribution, we generated a graph with the semi-log plot of the negative logarithm of the fraction of negative wells as compared to cell plating density (Figure 8A). The Poisson distribution line intercepts x at the reciprocal negative logarithm 1/e or 37% and defines μ=1, which is linearly proportional to the mean number of sphere-forming cells. This analysis showed that the cells of the neurosphere that were capable of forming new neurospheres was 1 in 800 as compared to the PRPs which was 1 in >1000 cells. Next we grew the cells as bulk cultures and analyzed self-renewal across serial passages (Figure 8B). Across the first two passages there was an amplification of cells that formed spheroids. Approximately 1% of the total cell population directly isolated from the neonatal SVZ formed spheroids whereas 4.3% of the cells in the 2nd passage formed secondary spheroids. However, from passage 3 through passage 4 (tertiary to quaternary) the percentage of self-renewing cells within the spheroids plummeted. Cells able to form tertiary spheroids generated in the 3rd passage declined to 3.5% of the total cell population and at the 4th passage less than 0.1% of the total cell population formed spheroids. By contrast, cells that formed neurospheres directly from the neonatal SVZ represented 0.7% of the total cell population, which increased to approximately 3% at the 2nd passage and then was maintained. Furthermore, primary neurospheres and spheroids were morphological dissimilar. Prior to the 1st passage the neurospheres (Figure 8B1) were more circular than the spheroids (Figure 8B2). Secondary neurospheres (Figure 8B3) and spheroids (Figure 8B4) were more similar. After three serial passages, the neurospheres consistently formed new symmetrical spheres (Figure 8B5), whereas the spheroids were extremely small and irregularly shaped (Figure 8B6). Progenitors exist outside the SVZ that can generate new glial cells (and neurons under non-physiological conditions) throughout adulthood; therefore, we evaluated the capacity of neocortical progenitors to generate spheroids and assessed their potentiality (Figure 9). Single-cell suspensions from neonatal and adult neocortices were grown in B104 CM. The neonatal neocortical cells (Figures 9A1 and 9A2) formed spheroids by 7 days while the adult neocortical cells (Figures 9B1 and 9B2) required twice the amount of time to produce spheroids. Surprisingly, progenitors in the adult neocortex only produced spheroids when the cells were grown in 2% O_2_, unlike the neonatal cells that grew well in more highly oxygenated medium. The cells from the adult brain also required a higher plating density to produce spheroids (3×10^5^ cells/ml). Next we compared the differentiation potentialities of neocortical versus SVZ PRPs from both neonates and adults. The differentiation patterns of the SVZ cells observed from the adult brain were not very different from the neonatal brain. Moreover, spheroids from the adult and neonatal neocortices produced both O4+ oligodendrocytes and GFAP+ astrocytes (Figures 9C1 and 9C2). By contrast progenitors isolated from the adult neocortex did not produce neurons. Finally, we assessed the relative numbers of PRPs present across the lifespan. With age the number of spheroids produced from the neocortex decreased (Figure 9D). By contrast, the capacity of the SVZ to produce spheroids was maintained.

## DISCUSSION

The SVZ is a germinal matrix that contains NSCs and progenitors of various potentialities. These stem and progenitor cells are present in the brain throughout adult life. Evidence has emerged to suggest that a PDGFRα+ precursor, with similar potentiality to the NSCs, also resides in this niche (Jackson et al., 2006). Whereas previous studies on the PDGFRα+ cells of the brain have focused on their roles in gliogenesis (Richardson et al., 1988; McKinnon et al., 2005), recent *in vivo* studies of the PRPs in the embryo and adult have implicated them in neurogenesis (Chojnacki and Weiss, 2004; Chojnacki et al., 2008; Rivers et al., 2008; Guo et al., 2010). The aim of this study was to establish a well defined *in vitro* culture system to study the PRPs of the postnatal SVZ, to determine whether the postnatal PRPs are stem cells or progenitors, to assay whether they are a homogeneous or heterogeneous population and to evaluate their relative abundance across the lifespan.

PDGF-AA is a growth factor that signals through PDGFRα to stimulate cell proliferation and to also promote cell survival (Barres et al., 1992; McKinnon et al., 2005). We modified the well-established neurosphere procedure to amplify the PRPs of the postnatal SVZ. By culturing isolated SVZ cells in media supplemented with PDGF-AA we obtained colonies of nonadherent cells that grew as clusters. We refer to these clusters as spheroids to distinguish them from neurospheres formed in EGF/FGF-2 supplemented media. Morphologically, primary spheroids have an asymmetric appearance versus primary neurospheres that grow as tight symmetric spheres.

Since PDGFRα is known to be associated with glial differentiation it seemed likely that the SVZ cells that were PDGF-AA-responsive would only produce glial progeny (Hill et al., 2013). However, we found that PRPs of the SVZ were not only a prominent source for forebrain macroglia, giving rise to oligodendrocytes and two types of astrocytes, but that they also produced neurons. Neurons are not traditionally thought to develop from PDGFRα+ precursors. Rather, they have been shown to develop from EGFR+ or FGFR+ precursors of the VZ and SVZ. However, our experiments clearly demonstrate that PDGFRα+ SVZ cells can give rise to immature neurons at least until postnatal day 12. Using FACS, we have recently established that Lex+/CD133−/NG2+/PDGFRα+ cells sorted from the neonatal SVZ and from neurospheres are capable of differentiating into A2B5−/GD3−/GFAP+ polygonal astrocytes, A2B5−/GD3+/GFAP+ radial glial-like astrocytes, TuJ1+ immature neurons and O4+ oligodendrocytes when cultured in the presence of PDGF-AA. Thus quatropotential progenitors also exist in the mouse SVZ (Buono et al., 2012). Moreover, our studies on the composition of the mouse SVZ confirmed that the multipotential PRPs are lost as the brain matures (Buono et al., 2012). Other laboratories have used transgenic animals and traced the fates of NG2+/PDGFRα+ cells throughout the adult CNS and discovered that these cells are capable of giving rise to new projection neurons in the ventral forebrain and in the piriform cortex (Rivers et al., 2008). More specifically NG2+/PDGFRα+ cells have been shown to differentiate into functional pyramidal glutamatergic neurons in the piriform cortex (Guo et al., 2010).

Like the SVZ, spheroids produced multiple cell types as assessed using a panel of markers that identified immature oligodendrocytes, type 1 astrocytes, type 2 astrocytes and neurons. Surprisingly a majority of spheroids analyzed in the B104 CM and PDGF-AA supplemented media produced spheroids that were capable of generating oligodendrocytes, astrocytes and neurons at the same ratio as the neurospheres. We initially hypothesized that our culture conditions would be optimal for growing glial lineage restricted progenitors but our results contradicted that hypothesis. Not only were these PRPs tripotential, making neurons, type 1 astrocytes and oligodendrocytes, but they were also capable of generating type 2 astrocytes. An important conclusion of our studies is that there is a unique, quatropotential progenitor within the SVZ.

Despite multiple attempts, we were unable to culture single PRPs to study their differentiation potentials. Therefore, to study single PRPs we used replication-incompetent retroviral fate mapping. In these studies, over 96% of the virally infected colonies contained identifiable glial cells. The majority of the colonies (65%) only generated astrocytes and 20% contained only oligodendrocytes. A third subset, approximately 10% of the progenitors, produced both oligodendrocytes and protoplasmic astrocytes. A small fraction of the infected cells (3–4%) did not stain for either GFAP or O4. These clones were likely comprised of progenitors that had not yet acquired lineage-restricted markers. Clusters of neurons and glia were not observed.

Since subsets of neurons, and striatal neurons in particular, can silence retroviral promoters (Gaiano et al., 1999), we performed fate-mapping studies using mixtures of GFP+ and GFP− PRPs. At the ratio analyzed for these studies, GFP+ cells comprised only 0.6% of the total population, enabling us to assess clonality. Many of the resulting spheroids were a mixture of GFP+ and GFP− cells, which is consistent with previous studies that showed that precursors can adhere to each other to form spheres and that smaller spheres can aggregate and eventually fuse to become a single larger sphere (Ladiwala et al., 2012). While spheroid fusion can complicate clonal analyses, the results of the hypergeometric probability model, using parameters matching our experimental setup, indicate that there is a less than 5% probability that two or more GFP+ cells contributed to a single spheroid even when 15 cells contributed to the formation of a spheroid. Our limited dilution studies indicate that this overestimates the number of spheroid forming cells, thus the probability that two or more GFP+ cells formed a spheroid is even less.

In the PRP mixing experiments, 51% of the colonies were astrocyte-generating, 21% were oligodendrocyte-generating, 3% were neuron-generating, 17% were bipotential astrocyte and oligodendrocyte-generating, 3% were bipotential astrocyte and neuron-generating, none produced oligodendrocytes and neurons and 4% were tripotential, generating astrocytes, oligodendrocytes and neurons. Thus, using two different approaches for lineage analyses, we have determined that the PRPs are very heterogeneous with unipotential, bipotential, tripotential and presumably quatropotential progenitors capable of generating oligodendrocytes and two types of astrocytes, as well as a subset of neurons. Moreover, our results show that the PRPs of the SVZ retain their proliferative and multi-potentiality into adulthood whereas PRPs in the neocortex become depleted and lineage restricted. The adult neocortical spheroids did not produce neurons, unlike the spheroids from the neonatal neocortex. This suggests that the PRPs in the adult neocortex, although capable of proliferating, are glial lineage restricted and thus different from the PRPs of the neonate. There is a controversy over whether some NSCs are PDGFRα+ (Jackson et al., 2006; Chojnacki et al., 2011). We show that the neurospheres and the spheroids have similar differentiation potentials but the PRPs have limited self-renewal capacity. After four serial passages, the neurosphere-forming cells retained their ability to form subsequent spheres, however, the spheroid-forming cells after repeated passages became depleted. Previous studies on mouse PRPs found that these cells could be passaged when FGF-2 was added to the media (Chojnacki and Weiss, 2004). However, given that other studies have shown that FGF-2 de-differentiates cells *in vitro* we did not add FGF-2 to our medium (Kondo and Raff, 2000). Moreover, in our experiments, rat PRPs could be grown as spheroids and passaged without FGF-2 for up to three generations. Therefore, PRPs, and even the quatropotential PRPs, appear to have a limited self-renewal capacity and, therefore, by definition are not stem cells. These data confirm and extend the work of Chojnacki and colleagues (Chojnacki and Weiss, 2004; Chojnacki et al., 2011).

Spheroids formed at low plating densities, supporting the view that a spheroid can be derived from a single PDGF-AA responsive precursor. However, the spheroids grew more robustly at high densities, and providing conditioned media from high density spheroid cultures restored the growth potential of the low density plated cells. These observations suggest that these PRPs secrete factors that exert autocrine and/or paracrine signaling effects. Although the exact mechanism is unknown, it has been suggested that growth factor-dependent SHH (sonic hedgehog) signaling promotes survival, growth and/or self-renewal of PRPs (Chojnacki and Weiss, 2004). When PRPs were grown in the presence of cyclopamine, an inhibitor of SHH signaling, colony size and number were reduced. Conversely, when the PRPs were grown in the presence of SHH their numbers increased. These data suggest that SHH is promoting the ability of PRPs to produce spheroids.

The detection of increased levels of phosphorylated ERK-1 and 2 after PDGF-AA stimulation provides direct evidence that these cells express functional PDGF receptors. Although spheroids that were constantly grown in PDGF-AA did express low levels of the PDGF receptors, they responded poorly to PDGF stimulation with no significant change in downstream targets molecules (data not shown). Since it has been well established that cells down-regulate growth factor receptors in the constant presence of growth factors, we removed the insulin and PDGF-AA in the medium for 6 h prior to stimulating the cells with PDGF-AA and collecting protein for Western blot analyses. Using this modified culture paradigm we were able to detect both PDGFRα and PDGFRβ and demonstrated that ERK-1 and 2 phosphorylation increased upon PDGF stimulation.

Despite the fact that the majority of cells within the spheroids were PSA-NCAM+, the spheroids were clearly comprised of a mixed population of cells that expressed markers of both restricted and unrestricted progenitors. Several markers of tripotential progenitors, such as PSA-NCAM (Grinspan and Franceschini, 1995; Ben-Hur et al., 1998), Dlx2 (Marshall and Goldman, 2002), Olig2 (Marshall et al., 2005) and NG2 (Nishiyama et al., 1996; Aguirre et al., 2004) were expressed by a large percentage of the cells. Almost 35% of the cells also expressed nestin, which is commonly associated with stem cells and early progenitors in the CNS (Dahlstrand et al., 1995; Kawaguchi et al., 2001; Beech et al., 2004; Mignone et al., 2004). Vimentin, another intermediate filament, which is expressed by immature astrocytes and glial progenitors was also present, but in a smaller subset of cells. Furthermore, ~20% of the cells were A2B5+, which is often used as a marker for glial-restricted precursors, but which was originally described as a neuronal maker (Eisenbarth et al., 1979; Rao and Mayer-Proschel, 1997; Scolding et al., 1999). GFAP was found in ~20% of the cells. Ganglioside GD3 and acetylated ganglioside GD3, both of which have been well established to be enriched on SVZ and cerebral progenitors (Levine et al., 1984; LeVine and Goldman, 1988) were present in a small percentage of the cells. This was unexpected given that GD3 is highly expressed in the *in vivo* neonatal SVZ.

Since an *in vitro* culture system has not been well established to grow PRPs of the postnatal SVZ, we evaluated combinations of B104 CM and PDGF-AA±noggin, as these growth supplements have been used to propagate neural precursors (Noble et al., 1988; Grinspan et al., 1990b; Gard and Pfeiffer, 1993; Grinspan and Franceschini, 1995; Avellana-Adalid et al., 1996; Nishiyama et al., 1996; Seidman et al., 1997; Zhang et al., 1998; Vitry et al., 1999; Lim et al., 2000; Kondo and Raff, 2004; Izrael et al., 2007). We found that these culture media formulations all generated ~80% PSA-NCAM+ cells, where PSA-NCAM is a marker established to be expressed by both precursors capable of producing neurons, astrocytes and oligodendrocytes. There was no significance between the individual conditions. These *in vitro* results support previous *in vivo* studies of postnatal SVZ-derived precursors, where ~80% expressed PSA-NCAM (Levison and Goldman, 1997). Spheroids produced in medium supplemented to 30% B104 CM generated the most equivalent balance between astrocytes and oligodendrocytes, while maintaining the highest percentage of GFAP−/O4− cells, which are likely non-lineage-restricted progenitors.

Spheroids grew larger when maintained in 2% O_2_ versus 20% O_2_. This observation is analogous to previous studies that showed that O_2_ tension is a critical determinant in NSC propagation (Chen et al., 2007; Pistollato et al., 2007; Le Belle et al., 2011). These studies also showed that growth in 5% O_2_ expanded nestin+/CD133+/CD24+ precursors that readily differentiated into all three neural lineages, whereas neural precursors maintained in 20% O_2_ preferentially generated astrocytes regardless of the presence of other mitogens (Chen et al., 2007; Pistollato et al., 2007). They also found that increases in O_2_ tension promoted oligodendrocyte maturation, which is similar to our findings. However, we did not observe the ratio of oligodendrocytes to astrocytes being affected by the concentration of O_2_, likely because our studies were performed on rat progenitors which are less sensitive to O_2_ than either mouse or human neural precursors (Chen et al., 2007; Pistollato et al., 2007).

Adult PRPs have been postulated to be tumor-initiating cells in the adult neocortex since they are slowly dividing cells that can produce both astrocytes and oligodendrocytes and persist across the lifespan (Assanah et al., 2009). Although gliomas are known to start from a single transformed cell and they are most frequently found in the adult neocortex, our data suggest that some tumor-initiating cells might also reside within the SVZ; however, were one of the SVZ PRPs to become transformed, it would more likely form a primitive neuroectodermal tumor rather than a glioma.
